# Temporary Anchorage Device: An Epitome of Anchorage in Orthodontic Treatment

**DOI:** 10.5005/jp-journals-10005-1099

**Published:** 2010-04-15

**Authors:** Preeth Shetty, US Krishna Nayak, Amitha M Hegde, Mary Jacob

**Affiliations:** 1 Reader, Department of Pedodontics and Preventive Children Dentistry, AB Shetty Memorial Institute of Dental Sciences Mangalore, Karnataka, India; 2 Professor and Head, Department of Orthodontics and Dentofacial Orthopedics, AB Shetty Memorial Institute of Dental Sciences Mangalore, Karnataka, India; 3 Professor and Head, Department of Pedodontics and Preventive Children Dentistry, AB Shetty Memorial Institute of Dental Sciences Mangalore, Karnataka, India; 4 Postgraduate Student, Department of Pedodontics and Preventive Children Dentistry, AB Shetty Memorial Institute of Dental Sciences Mangalore, Karnataka, India

**Keywords:** Anchorage, Miniscrew, Palatal implant, Temporary anchorage device.

## Abstract

One of the most important phases of oral health is the form and function of the oral mechanism. Recently, pediatric dentists are concerned with the obvious esthetic disabilities and the pathologic implications of the malposed teeth. Interceptive and functional orthodontic treatment is playing a major role in these discrepancies. Anchorage is an important consideration in orthodontics, particularly if force is applied entirely to the teeth. For many years, clinicians have searched for a form of anchorage that does not rely on patient cooperation. During the last few decades, a wealth of new information has accumulated to such an extent that the present authors thought it appropriate to let these advances make an impact by suggesting a revised definition and classification of anchorage. This paper also gives a brief insight on evolution of anchorage and its application in pediatric dentistry.

## INTRODUCTION

Anchorage is a challenging aspect of orthodontic treatment. It is best described by the famous quote from the Greek philosopher Archimedes, “Give me a place to stand and I will move the earth”. This Greek mathematician and writer on science and mechanics, presents the same problem, on a vastly greater scale than the clinicians face today. This is the problem of adequate anchorage. Anchorage has been defined as the source of resistance to the forces generated in reaction to the active components of an appliance. In simple words, it means resistance to displacement. Every orthodontic appliance consists of two elements: An active (tooth movement) and resistance element (anchorage) that makes tooth movement possible. Anchorage can be related to Newton’s third law, therefore, all anchorage is relative and all resistance is comparative. The importance of anchorage is more keenly appreciated when it has been neglected. Anchorage loss may jeopardize a successful result because of inappropriate movement of the anchor teeth which results in insufficient space remaining to achieve the intended tooth movements. Conventional anchorage methods generally rely on patient compliance and the result is unwanted reciprocal tooth movements. In an effort to overcome some of these problems, skeletal anchorage has been increasingly incorporated into orthodontic treatment for over 25 years.^1^ This literature tries to redefine the terminology of anchorage, classification and gives an insight on the journey of quest for absolute anchorage.

## DEFINITION

McCoy’s definition of anchorage should be stated here for it does not picture intraoral anchorage as omnipotent, but relies upon the operator’s judgment to select proper anchorage for the tooth movement which he may wish to accomplish. He stated, “anchorage consists in the selection of adequate and properly distributed resistance units for the control and direction of force applied to the teeth, for arch development or for lesser tooth movements”.

Graber^3^ said, “it is the nature and degree of resistance offered by an anatomic unit for the purpose of effecting tooth movement”.

With the evolution of absolute anchorage from tooth borne and tissue borne, there is a need to redefine the terminology as―it is the nature and degree of resistance in anterior-posterior, transverse and vertical planes offered by an anatomic or non-anatomic unit or units for the purpose of effecting the tooth movement.

## CLASSIFICATION OF ANCHORAGE

Anchorage has been classified and named in several ways. Here, we have tried to put forth all the available types of anchorage and classified them (Table 1). Anchorage can be derived from various structures both intraoral and extraoral (e.g. action of muscles,^4^ craniofacial bones, etc). Cortical bone is more resistant to resorption, and tooth movement is slowed when a root contacts it. Anchorage control is different in cortical^5^ as compared with the medullary bone. It has long been realized that if structures other than the teeth could be made to serve as anchorage, it would be possible to produce tooth movement or growth modification without unwanted side effects. With the development of successful bone implant techniques^6^ the potential existed, for what could be described as absolute anchorage, with no tooth movement except what was desired. Temporary anchorage devices^7^ (TADs) are a relatively recent addition to orthodontic anchorage. These are immediately loaded miniscrews and osseointegrated palatal implants that are placed to control tooth movement during orthodontic treatment and removed when the treatment is completed.

## ANCHORAGE IN THREE MILLENNIA

Weinberger,^8^ in his book, stated that the first important appliance that marks a distinct step was given by Pierre Fauchard in 1723. The chief function of this appliance was to expand the arch. It has been known as a band, bow, bandelette and short and long band, but in reality, it was the earliest form of the expansion arch as we know it today. Delabarre, according to Weinberger, devised the first wire crib which was later to prove very useful as an anchorage. He also described for the first time the “metallic box” or orthodontic band, as we now term it.

**Table 1:** **Table 1:** Anchorage: Revised version of classification

Type of force		• Simple	
		• Stationary	
		• Reciprocal	
		• Reinforced	
Site of anchorage		• Intraoral	
		– Intramaxillary	
		– Intermaxillary	
		• Extraoral	
		– Cervical	
		– Occipital	
		– Cranial	
		– Facial	
		• Muscular^3^	
		• Cortical^4^	
		• Skeletal/absolute^4^	
		– Osseointegrated dental	
		implants^5^	
		– Direct	
		– Indirect	
		– Temporary anchorage	
		devices^6^(TAD)	
No. of anchorage units		• Single/primary	
		• Compound	
		• Reinforced	

One of the earliest accounts of the slipping of anchorage units, Weinberger tells, was reported by Kniesel in 1836. He condemned the method of rotating teeth as was formerly recommended by Geraudly and others because of a lack of anchorage. He stated, “an adjoining tooth or several of them must serve as a fulcrum for the tooth. By this means the pressure of the ligature is divided evenly in an entire circle, then, according to the laws of the mechanics, the tooth that serves as a fulcrum is pushed just as much out of its position, as the distorted tooth”.

Clinical experience continued to be a good teacher and gradually from these early appliances began to evolve the efficient orthodontic mechanisms as we know today. With each step in this evolution, researchers seemed to become more and more mindful of the fact that the teeth could not be considered as anchorage themselves, but were secured to a base which, though strong and unyielding, was capable of undergoing changes in form.

Most writers divide anchorage into two types: Intra-oral anchorage and extraoral anchorage. As clinicians, we must use our judgment in the selection and utilization of anchorage, no matter what mechanism or technique we employ. Often it is found that no matter what principles we employ in securing proper anchorage, the desired tooth movement cannot be obtained with intraoral resistance units alone. The answer to such a problem can only be the use of extraoral anchorage which had been overlooked by many clinicians.^9^

Headgear, elastics, adjacent teeth and many appliances have been suggested as anchorage, however, the main drawback was that most relied on patient compliance to be successful.^10^ Recently, many incidents have made pediatric dentists aware of safety hazards surrounding the use of extraoral appliances like facebow, low and high pull head gear. They have been found to be a source of potential injury to the children, since such appliances have been designed in the same principle of a slingshot. The ends of the facebow also may pose serious damage to the inner mouth or tongue or even lips and eyes of a child when pulled out sufficiently.^11,12^

For many years, clinicians have searched for a form of anchorage that does not rely on patient cooperation, although the answer already lay in the implant, dentists used to replace missing teeth and oral surgeons used to hold bone segments together. Now these divergent lines have come together in the form of stationary anchorage. Earlier it was thought that osseointegration in implants was necessary for orthodontic anchorage.^13-15^ Experimental biomechanic studies,^16^ studies on animal models^17,18^ and clinical investigations^19^ have shown that dental implants placed in the alveolar bone are resistant to orthodontic force application.

Although endosseous implants, miniplates and onplants have been used successfully for orthodontic anchorage and reinforcing anchorage in comparison with extraoral anchorage, their clinical applications are limited because of their size and complicated fixture designs. Other problems, like long waiting period (2-6 months) for bone healing and osseo-integration, comprehensive surgical procedures and difficult removal after treatment, make the children uncooperative during the entire treatment phase.^20^

Kanomi^21^ first mentioned a temporarily placed mini-screw for orthodontic anchorage. The following years brought more refined screw designs.^22^ Temporary anchorage devices^7,20^ (TAD), like miniplates, miniscrews, microscrews, microimplants are simpler alternatives to endosseous implants and onplants and their advantages include smaller size, greater number of implant sites, simpler surgical placement without any full flap retraction, immediate loading without any need for laboratory work, easier removal after treatment and lower cost. Stability of the dental implants relies on osseointegration which requires several weeks for the host bone to achieve intimate contact with the implant.^23^ Generally, miniscrews are loaded sooner. Histological studies indicated that small titanium screws can function as rigid osseous anchorage against orthodontic loads with a minimal healing period.^24,25^ Romanos et al^24^ showed that immediate loading increased the ossification of the alveolar bone around the implant.

Success rates of TADs were higher in maxilla than in mandible and researchers significantly supported the maxilla as a more suitable placement site for miniscrew.^26-28^ There are preferable areas that offer sufficient bone and root distances. Poggio et al^29^ evaluated tomographic images of mandible and maxilla to define “safe zones” for placing miniscrews. In the maxilla, they recommended inter-radicular spaces between the canine and the second molar on the palatal side, and between the canine and the first molar on the buccal side. In the mandible, they suggested inter-radicular spaces between the canine and the second molar. TADs have been placed in the midpalatal suture area of adults, and the parapalatal area in adolescents to prevent possible developmental disturbances of the midpalatal sutures.^30^ The midpalatal area within 1mm of the midsagittal suture is composed of the thickest bone available in the whole palate, and the thickness of the soft tissues in the midpalatal area is uniformly 1 mm posterior to the incisive papilla, ensuring biomechanical stability of the miniscrews. ^31,32^ Gedrange et al^33^ concluded from a study on human cadavers that the quality of placement and bone structure are more important than the length of the orthodontic implant for implant stability.^34^

## PEDIATRIC DENTIST’s PERCEPTION

Interceptive orthodontic between ages 3 to 8 uses a special window-of-time-opportunity to make space for adult teeth. Making space early may reduce or eliminate the need for further orthodontic treatment, tooth extraction surgery and even jaw surgery at a later age. Early orthodontic treatment is now more generally accepted, as a means of gaining the greatest possible control over form and function and changes with time. It seems logical to assume that certain problems should be treated early to take advantage of the most cra-niofacial growth. Many difficult orthodontic problems reside in maldeveloped and/or malrelated skeletal structures. Although interceptive orthodontic procedures often do not produce finished orthodontic results without a second phase of treatment in the permanent dentition, several studies have suggested that systematically planned interceptive treatment in the mixed dentition might contribute to a significant reduction in treatment need between the ages of 8 and 12 years, often producing results so that further need can be categorized as elective.^35^ Therefore, from a pediatric dentist’s point of view, to overcome the safety hazards surrounding the use of extraoral appliances, esthetic concerns and patient cooperation, skeletal anchorage provides an edge over others. Studies have shown both implants and headgears proved to be effective methods of reinforcing anchorage. In comparison with headgears, all TADs are easier to use, more socially acceptable for children and the period of activation is shorter because they apply continuous forces.^36^

TADs have the potential to provide some kind of anchorage, which enables orthodontic tooth movements that might be impossible with conventional anchorage methods. Satisfactory orthopedic effects have been achieved in growing children especially for autorotation of mandible due to vertical manipulations of buccal segments and in combination with extraoral or intermaxillary forces. Palatal implants have been found effective in reinforcing extraoral anchorage with headgear. Pediatric dentists should now deploy this potent anchorage reinforcing in TADs as an effective modality in achieving good orthopedic and orthodontic results. In conclusion, the use of TADs really expands the envelope of discrepancies in which orthodontic treatment might be successful. However, the relative effectiveness and efficiency of all different TADs used for various clinical situations need to be evaluated further in terms of their application toward orthopedic force applications in pediatric patients.
